# Supplementary figures and images for: Mapping global kimberlite potential from reconstructions of mantle flow over the past billion years
Source: PLoS One. 2022 Jun 9;17(6):e0268066. doi: 10.1371/journal.pone.0268066 (PMC9182341; doi:10.1371/journal.pone.0268066)

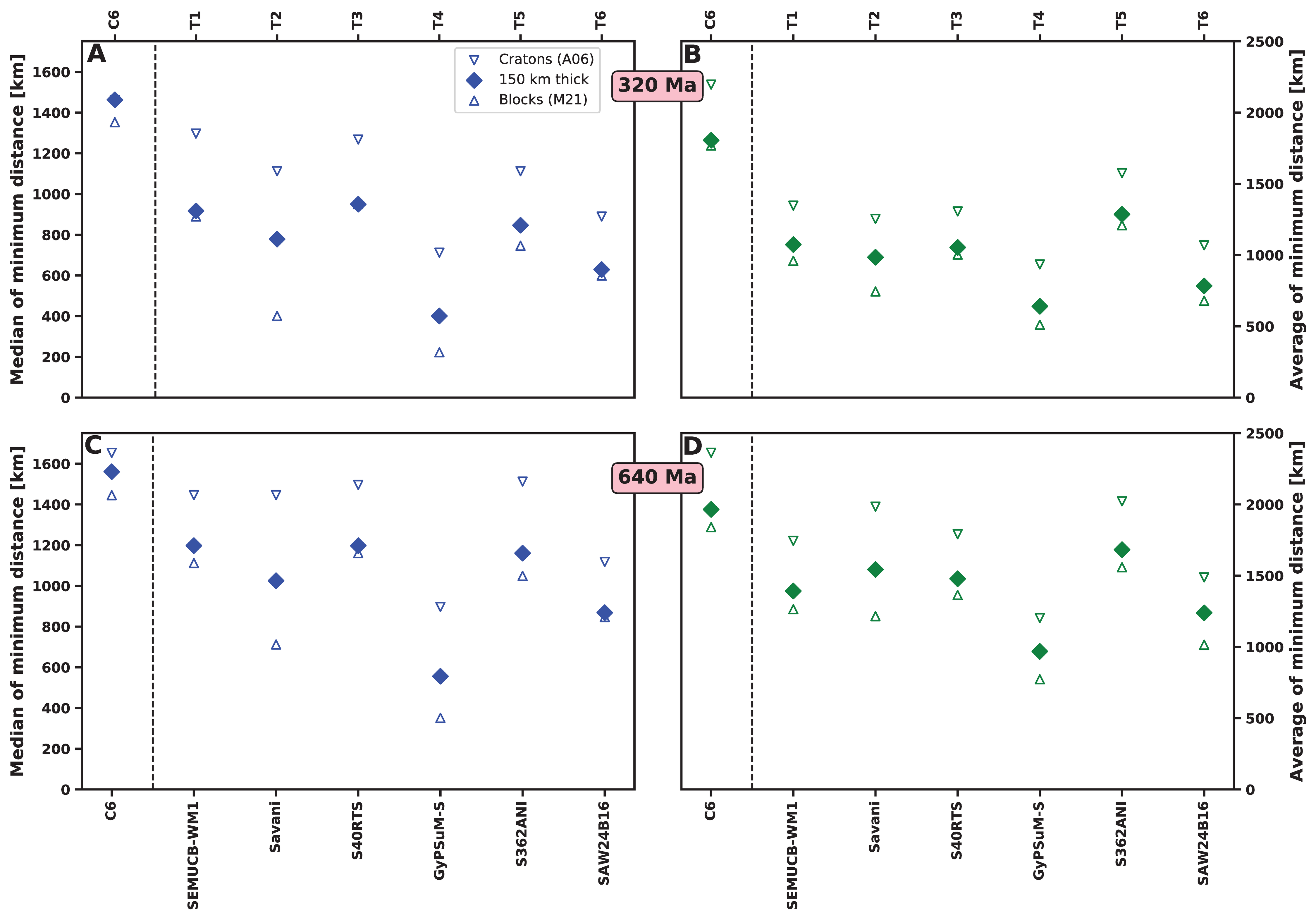

Supplement: S1 Fig — (TIF) [file pone.0268066.s005.tif]
