# Supplementary material for: Mapping global kimberlite potential from reconstructions of mantle flow over the past billion years
Source: PLoS One. 2022 Jun 9;17(6):e0268066. doi: 10.1371/journal.pone.0268066 (PMC9182341; doi:10.1371/journal.pone.0268066)
Supplement: Attachment — Submitted filename: PONE-D-21-39227_reviewer_mod.pdf [file pone.0268066.s006.pdf]

PLOS ONE

Global kimberlite prospectivity from reconstructions of mantle flow over the past billion years

--Manuscript Draft--

**Manuscript Number:**	PONE-D-21-39227
**Article Type:**	Research Article
**Full Title:**	Global kimberlite prospectivity from reconstructions of mantle flow over the past billion years
**Short Title:**	Global kimberlite prospectivity from reconstructions of past mantle flow
**Corresponding Author:**	Nicolas Flament<br>University of Wollongong<br>Wollongong, AUSTRALIA
**Keywords:**	tectonics; mantle convection; resources exploration; kimberlite; diamond
**Abstract:**	Kimberlites are the primary source of economic grade diamonds. Their geologically rapid eruptions preferentially occur near thick Archean cratonic lithosphere. Studies combining tomographic models with tectonic reconstructions and kimberlite databases have revealed spatial correlations between Large Low Shear Velocity Provinces in the lowermost mantle and reconstructed global kimberlite eruption locations over the last 320 Myr. These spatial correlations assume that the lowermost mantle structure has not changed over time, which is at odds with mantle flow models that show basal thermochemical structures to be mobile features shaped by cold sinking oceanic lithosphere.<br>Here we investigate the match to the global kimberlite record of stationary seismically slow basal mantle structures (as imaged through tomographic modelling) and mobile hot basal structures (as predicted by reconstructions of mantle flow over the past billion years). We refer to these structures as “basal mantle structures” and consider their intersection with reconstructed cratons to represent areas with a high potential for past eruptions of kimberlites, and therefore areas of potential interest for diamond exploration. We use the distance between the reconstructed kimberlite eruption locations and model high-prospectivity areas as an indicator of model success, and we find that mobile lowermost mantle structures are as close to reconstructed kimberlites as stationary ones. Additionally, we find that mobile lowermost mantle structures better fit major kimberlitic events, such as the South African kimberlite bloom around 100 Ma and the eastern Canadian kimberlite record. Mobile basal structures are therefore consistent with both solid Earth dynamics and with the kimberlite record.
**Order of Authors:**	Anton Grabreck
	Nicolas Flament
	Ömer F Bodur
**Additional Information:**	
**Question**	**Response**
**Financial Disclosure**<br><br>Enter a financial disclosure statement that describes the sources of funding for the work included in this submission. Review the submission guidelines for detailed requirements. View published research articles from *PLOS ONE* for specific examples.<br><br>This statement is required for submission	NF and OFB were supported by Australian Research Council (https://www.arc.gov.au/) grant LP170100863.

Powered by Editorial Manager® and ProduXion Manager® from Aries Systems Corporation

and **will appear in the published article** if the submission is accepted. Please make sure it is accurate.<br><br>**Unfunded studies**<br>Enter: *The author(s) received no specific funding for this work.*<br><br>**Funded studies**<br>Enter a statement with the following details:<br>• Initials of the authors who received each award<br>• Grant numbers awarded to each author<br>• The full name of each funder<br>• URL of each funder website<br>• Did the sponsors or funders play any role in the study design, data collection and analysis, decision to publish, or preparation of the manuscript?<br>• **NO** - Include this sentence at the end of your statement: *The funders had no role in study design, data collection and analysis, decision to publish, or preparation of the manuscript.*<br>• **YES** - Specify the role(s) played.<br><br>* typeset	
**Competing Interests**<br><br>Use the instructions below to enter a competing interest statement for this submission. On behalf of all authors, disclose any competing interests that could be perceived to bias this work—acknowledging all financial support and any other relevant financial or non-financial competing interests.<br><br>This statement is required for submission and **will appear in the published article** if the submission is accepted. Please make sure it is accurate and that any funding sources listed in your Funding Information later in the submission form are also declared in your Financial Disclosure statement.<br><br>View published research articles from *PLOS ONE* for specific examples.	The authors have declared that no competing interests exist.

Powered by Editorial Manager® and ProduXion Manager® from Aries Systems Corporation

**NO authors have competing interests**<br><br>Enter: *The authors have declared that no competing interests exist.*<br><br>**Authors with competing interests**<br><br>Enter competing interest details beginning with this statement:<br><br>*I have read the journal's policy and the authors of this manuscript have the following competing interests: [insert competing interests here]*<br><br>* typeset	
**Ethics Statement**<br><br>Enter an ethics statement for this submission. This statement is required if the study involved:<br><br>• Human participants<br>• Human specimens or tissue<br>• Vertebrate animals or cephalopods<br>• Vertebrate embryos or tissues<br>• Field research<br><br>Write "N/A" if the submission does not require an ethics statement.<br><br>General guidance is provided below. Consult the submission guidelines for detailed instructions. **Make sure that all information entered here is included in the Methods section of the manuscript.**	N/A

Powered by Editorial Manager® and ProduXion Manager® from Aries Systems Corporation

**Format for specific study types**<br><br>**Human Subject Research (involving human participants and/or tissue)**<br>• Give the name of the institutional review board or ethics committee that approved the study<br>• Include the approval number and/or a statement indicating approval of this research<br>• Indicate the form of consent obtained (written/oral) or the reason that consent was not obtained (e.g. the data were analyzed anonymously)<br><br>**Animal Research (involving vertebrate animals, embryos or tissues)**<br>• Provide the name of the Institutional Animal Care and Use Committee (IACUC) or other relevant ethics board that reviewed the study protocol, and indicate whether they approved this research or granted a formal waiver of ethical approval<br>• Include an approval number if one was obtained<br>• If the study involved *non-human primates*, add *additional details* about animal welfare and steps taken to ameliorate suffering<br>• If anesthesia, euthanasia, or any kind of animal sacrifice is part of the study, include briefly which substances and/or methods were applied<br><br>**Field Research**<br><br>Include the following details if this study involves the collection of plant, animal, or other materials from a natural setting:<br>• Field permit number<br>• Name of the institution or relevant body that granted permission	
**Data Availability**<br><br>Authors are required to make all data underlying the findings described fully available, without restriction, and from the time of publication. PLOS allows rare exceptions to address legal and ethical concerns. See the PLOS Data Policy and FAQ for detailed information.	Yes - all data are fully available without restriction

A Data Availability Statement describing where the data can be found is required at submission. Your answers to this question constitute the Data Availability Statement and **will be published in the article**, if accepted.<br><br>**Important:** Stating 'data available on request from the author' is not sufficient. If your data are only available upon request, select 'No' for the first question and explain your exceptional situation in the text box.<br><br>Do the authors confirm that all data underlying the findings described in their manuscript are fully available without restriction?	
**Describe where the data may be found in full sentences. If you are copying our sample text, replace any instances of XXX with the appropriate details.**<br><br>• If the data are **held or will be held in a public repository**, include URLs, accession numbers or DOIs. If this information will only be available after acceptance, indicate this by ticking the box below. For example: *All XXX files are available from the XXX database (accession number(s) XXX, XXX.).*<br>• If the data are all contained **within the manuscript and/or Supporting Information files**, enter the following: *All relevant data are within the manuscript and its Supporting Information files.*<br>• If neither of these applies but you are able to provide **details of access elsewhere**, with or without limitations, please do so. For example:<br><br>*Data cannot be shared publicly because of [XXX]. Data are available from the XXX Institutional Data Access / Ethics Committee (contact via XXX) for researchers who meet the criteria for access to confidential data.*<br><br>*The data underlying the results presented in the study are available from (include the name of the third party*	Key model results and scripts are available at https://doi.org/10.5281/zenodo.5760115.

and contact information or URL).<br>• This text is appropriate if the data are owned by a third party and authors do not have permission to share the data.<br><br>* typeset	
Additional data availability information:	

Manuscript

Click here to access/download;Manuscript;1_Manuscript.pdf

Global kimberlite prospectivity from reconstructions of mantle flow over the past billion years

Anton Grabreck[1], Nicolas Flament[1*] and Ömer F. Bodur[1]

[1]GeoQuEST Research Centre, School of Earth, Atmospheric and Life Sciences, University of Wollongong, Northfields Avenue, NSW 2522, Australia

*Corresponding author: nflament@uow.edu.au

Abstract

Kimberlites are the primary source of economic grade diamonds. Their geologically rapid eruptions preferentially occur near thick Archean cratonic lithosphere. Studies combining tomographic models with tectonic reconstructions and kimberlite ~~databases~~ have revealed spatial correlations between Large Low Shear Velocity Provinces in the lowermost mantle and reconstructed global kimberlite eruption locations over the last 320 Myr. These spatial correlations assume that the lowermost mantle structure has not changed over time, which is at odds with mantle flow models that show basal thermochemical structures to be mobile features shaped by cold sinking oceanic lithosphere.

Here we investigate the match to the global kimberlite record of stationary seismically slow basal mantle structures (as imaged through tomographic modelling) and mobile hot basal structures (as predicted by reconstructions of mantle flow over the past billion years). We refer to these structures as "basal mantle structures" and consider their intersection with reconstructed cratons to represent areas with a high potential for past eruptions of kimberlites, and therefore areas of potential interest for diamond exploration. We use the distance between the reconstructed kimberlite eruption locations and model high-prospectivity areas as an indicator of model success, and we find that mobile lowermost mantle structures are as close to reconstructed kimberlites as stationary ones. Additionally, we find that mobile lowermost mantle structures better fit major kimberlitic events, such as the South African kimberlite bloom around 100 Ma and the eastern Canadian kimberlite record. Mobile basal structures are therefore consistent with both solid Earth dynamics and with the kimberlite record.

1. Introduction

The majority of diamonds, including economic grade diamonds, were formed at the base of thick continental lithosphere [1]. Using xenolith data from kimberlitic eruptions, ref. [2] determined the temperature-pressure window that allows for diamond formation and stability as between 1200 ºC and 1570°C and above 5 GPa. "Clifford's rule" postulates that cratonic diamonds are formed in cratons with thick lithospheric keels, providing environments with sufficiently high pressures and low temperatures for diamond stability [3].

Cratons of Archean age are particularly thick [possibly up to ~300 km; e.g. 4] and are an ideal environment for diamond formation [5-8]. Kimberlite eruptions are rapid magmatic events with magma ~~erupting~~ at speeds up to 20 m s^{-1} [9, 10], which is geologically instantaneous compared to average plate motion at ~1.26 × 10^{-9} m s^{-1} (4 cm yr^{-1}) over the last 200 Myr [11]. The average depth of kimberlite eruptions is 200 km [3], although some rare superdeep (sub-lithospheric) kimberlites may have ascended from as deep as 800 km [12, 13]. The kimberlite record extends deep in geological times and is considered to be continuous from 2 Ga; the oldest known kimberlite ~~pipe~~ is 2.85 Gyr old [14]. Over 60% of known kimberlite pipes erupted during Mesozoic and Cenozoic times [15], which could be either the result of the preferential preservation of younger kimberlites or of increased eruption rate during the last 200 Myr [16]. Southern Africa underwent the highest kimberlite activity during this period, therefore this area preserves the most known kimberlite occurrences [17].

Combining databases of volcanic products with tectonic reconstructions and SMEAN [an average of three S-wave tomographic models; 18], ref. [7] showed that the reconstructed locations of the majority of Large Igneous Provinces (LIPs) and kimberlites since ~~320~~ Ma (to the exception of Canadian kimberlites) are within ~1,000 km of the edges of two large seismically slow provinces above the Core-Mantle Boundary (CMB) commonly known as

Large Low-Shear Velocity Provinces [LLSVPs; 19]. The LLSVPs, presently positioned under the Pacific Ocean (the Pacific LLSVP) and under the African continent (the African LLSVP), are ~7,500 km in radius and 500-1,000 km in height - together they cover up to 50% of the CMB [19, 20]. The low shear-wave velocity of LLSVPs indicates that these structures are hotter than surrounding ambient mantle, and gradients in seismic waves at the edges of LLSVPs [21] suggest that the structures are intrinsically denser than surrounding mantle, although the density contrast remains debated [e.g. 22].

Due to their spatial relationship with volcanic surface features, the regions along the edge of LLSVPs are sometimes called Plume Generation Zones (PGZs) [23]. The specific relationship between the edges of LLSVPs and reconstructed locations of volcanic products is debated, with studies showing that the relationship is instead between the interior of LLSVPs and reconstructed locations of volcanic products [24, 25]. It is generally accepted that major LIPs are linked to deep mantle plumes [26], and the synchronicity of Mesozoic-Cenozoic Kimberlite blooms with periods of LIP eruption suggests a potentially common heat source for kimberlite and LIP eruptions [7]. However, the physical process connecting kimberlite magmatism ~~[on average from ~ 200 km depth; 3]~~ to LLSVPs [between 2000 km and 2500 km depth; 19] remains to be established. The spatial link between LLSVPs and reconstructed kimberlites eruption locations implies that hot basal mantle structures could have been stationary and rigid over time [7]. In contrast, global geodynamic models have revealed that similar hot basal mantle structures can form in response to the history of surface plate motions and plate subduction [27, 28].

Here we assume that kimberlite eruptions are linked to mantle upwelling occurring above basal mantle structures, following ref. [7]. We use the global tectonic reconstruction of ref. [29] to derive boundary conditions for forward mantle convection models that predict the evolution of the structure of the mantle over the past billion years. We map the intersection of basal

mantle structures (either stationary from tomography or mobile from flow models) and reconstructed cratons, which represent areas with a high-prospectivity potential for past eruptions of kimberlites and are therefore of potential interest to diamond exploration. We use the distance between the reconstructed locations of known kimberlite eruptions and model high-prospectivity areas as an indicator of model success and evaluate the fit of different models to the kimberlite record for selected periods and regions of high kimberlite activity.

2. Datasets and methods

Our approach uses tectonic plate reconstructions to link past kimberlite eruption locations, thick continental lithosphere, and basal mantle structures either imaged by tomographic models or predicted by mantle flow models.

2.1. Tectonic reconstructions

We use the global tectonic reconstruction by ref. [29], which is the first continuous full-plate reconstruction for the last billion years. This tectonic reconstruction links full-plate reconstructions by ref. [30] from 1,000-520 Ma, by ref. [31, 2018] from 500 Ma to 410 Ma and by ref. [33] from 410 to 0 Ma. We consider the tectonic reconstruction in its original paleomagnetic reference frame (reconstruction hereafter referred to as M21), as well as in a reference frame in which the net rotation of the lithosphere with respect to the mantle was removed, which is more appropriate for mantle flow models [e.g. 34, 35]. This latter reconstruction is hereafter referred to as M21NNR ("No Net Rotation").

2.2. Kimberlite database and cratons

We use the kimberlite database of ref. [15] that contains 1,133 kimberlites (~ 20% of the known kimberlite record) selected to not over-represent more studied economic (diamondiferous) kimberlite clusters in order to better capture the evolution of kimberlite magmatism. Kimberlite occurrences were sampled at 20 Myr intervals to match the temporal resolution used for the output of global mantle flow models. For each age of interest a_n, kimberlite occurrences were selected if they erupted within the period a_n + 10 Myr < $a_n \leq a_n$ - 10 Myr.

The age of the lithosphere can be established locally based on xenoliths carried to the surface by kimberlites [e.g. 36], and continental scale maps require interpolation. We therefore consider two end-member sets of Archean craton geometries: regions with tectonic ages greater than 2.5 Ga in the TC1 model of ref. [37] and the much larger cratonic shapes of ref. [29], designed for the purpose of tectonic reconstructions. Almost all present-day locations of kimberlites fall within the craton shapes from ref. [29], however, this is not the case for the smaller shapes from ref. [37] (Fig. 1a). There is a peak in kimberlite magmatism at around 100 Ma (Fig. 1b) with significant number of kimberlite occurrences in southern Africa (Fig. 1a).

Figure 1. Distribution of cratons and kimberlite eruptions. A/ Outline of cratons considered in this study: brown polygons from ref. [29] and grey polygons from ref. [37]; and 957 kimberlite eruptions from ref. [15] since 640 Ma, sampled in 20 Myr increments and coloured by age. Coastlines are shown in grey. **B/** Histogram showing the temporal distributions of kimberlites occurrences from ref. [15] since 640 Ma.

2.3. Reconstructions of past global mantle flow

We reconstruct the evolution of mantle flow since one billion years ago using *CitcomS* [38] in which the mantle is a shell represented with finite-elements in spherical geometry. We use 129 × 129 × 65 × 12 ≈ 13 million elements to obtain an average resolution of ~50 × 50 × 15 km at the surface, ~40 x 40 x 100 km in the mid-mantle, and ~28 × 28 × 27 km at the core-mantle boundary (CMB). We use a version of *CitcomS* [39] modified to progressively assimilate the thermal structure of the lithosphere and of subducting slabs (to ~350 km depth) determined from the synthetic age of the ocean floor [40] as well as surface velocities obtained from tectonic reconstructions with continuously closing plate polygons [41]. These boundary conditions are assimilated in one-million-year intervals. This approach makes it possible to obtain one-sided subduction in time-dependent global mantle convection models with computationally affordable resolution and viscosity variations.

We use the extended-Boussinesq approximation, which accounts for viscous dissipation, an adiabatic temperature gradient, internal heating and a decrease in the coefficient of thermal expansion by a factor of two over the thickness of the mantle [42]. Convection vigour was determined by the Rayleigh number $Ra = (\alpha_0 \rho_0 g_0 \Delta T h_M^3)/(\kappa_0 \eta_0)$, where the subscript "0" indicates reference values, with α_0 the coefficient of thermal expansion, $\rho_0 =$ 4,000 kg m^{-3} is the density, $g_0 = 9.81$ m s^{-2} the gravitational acceleration, $\Delta T = 3{,}100$ K the temperature change across the mantle, $h_M = 2{,}867$ km the thickness of the mantle, $\kappa_0 = 1 \times 10^{-6}$ m^2 s^{-1} the thermal diffusivity and $\eta_0 = 1.1 \times 10^{21}$ Pa s is the viscosity. With the values listed above $Ra = 7.8 \times 10^7$. The dissipation number that controls shear heating is $Di = (\alpha_0 g_0 R_0)/C_{p_0}$, with R_0 = 6,371 km being the radius of Earth and C_{p_0}= 1200 J kg^{-1} K^{-1} the reference heat capacity *Di* = 1.56. Viscosity varies with depth, composition, temperature and pressure following $\eta = \eta(r)\, \eta_0\, \eta_C \exp\left\{\frac{[E_\eta + \rho_0 g Z_\eta (R_0 - r)]}{[R(T + T_{off})]} - \frac{[E_\eta + Z_\eta (R_0 - R_c)]}{[R(T_{\mathrm{CMB}} + T_{off})]}\right\}$, with $\eta(r) = 0.02$

above 160 km depth and between 310–660 km depth, $\eta(r) = 0.002$ between 160–310 km depth (asthenosphere), and $\eta(r) = 0.02$ below 670 km depth (lower mantle). The compositional viscosity pre-factor η_C was varied across model cases for the basal layer (see Table 1). r is the radius, $R_C = 3{,}504$ km is the radius of the core, $E_\eta = 275$ kJ mol^{-1} is the activation energy, $Z_\eta = 2.1 \times 10^{-6}$ m^3 mol^{-1} is the activation volume, $R = 8.31$ J mol^{-1} K^{-1} is the universal gas constant, T is the dimensional temperature, $T_{\text{off}} = 452$ K is a temperature offset and $T_{\text{CMB}} = 3380$ K is the temperature at the core-mantle boundary. The viscosity pre-factor, activation energy, activation volume and temperature offset were selected to obtain variations in viscosity over three orders of magnitude (viscosity variations were limited to the range 1.1×10^{20} Pa s - 2.2×10^{23} Pa s) across the range of temperatures and pressures (Fig. 2).

Table 1. Parameters varied across mantle flow models.

Case number	Reconstruction	Basal layer viscosity pre-factor	Initial slab depth (km)	Basal layer density $\delta\rho_b$ (%)
**C1**	M21NNR	10	1,000	+1.02
**C2**	M21NNR	10	1,000	**+1.43**
**C3**	M21NNR	**1**	**550**	**+2.05**
**C4**	M21NNR	**1**	**550**	+1.02
**C5**	M21NNR	10	**550**	+1.02
**C6**	**M21**	10	1,000	+1.02

Values differing from Case 1 are in bold.

Figure 2. Temperature and viscosity with depth. Horizontally averaged present-day mantle temperature (left-hand side) and viscosity (right-hand side) for Case 2. Solid lines show the average, and dashed lines show the minimum and maximum. The grey line on the right-hand side panel is a viscosity profile adjusted to fit the geoid and post-glacial rebound [43].

The initial condition at 1 Ga consists of an adiabatic temperature profile between two thermal boundary layers, with slabs inserted down to either 550 km or 1,000 km depth (Table 1) with a dip of 45º down to 425 km and a dip of 90º below 425 km depth. For cases with slabs inserted down to 1,000 km depth, slabs are twice as thick in the lower mantle than in the upper mantle to account for advective thickening in the more viscous lower mantle [44]. The basal thermal boundary layer is initially 225 km thick and includes a 113-km thick layer [2% of the volume of the mantle; 45] that is denser than ambient mantle. The density contrast between the basal material and ambient mantle, obtained using tracers, was varied from 1.02% to +2.05% across model cases (Table 1).

As slabs sink into the mantle, the initially homogenous basal thermochemical layer is deformed [28, 46] and forms basal mantle structures of changing area. To assess this change, we quantify the evolution of the fractional area (f_a) of high temperature clusters in the models; we also compare it to the area of slow-velocity clusters in tomographic models.

2.4. Imaging lower mantle structures through cluster analysis

We consider six global S-wave tomographic models that use variations in seismic wave velocities to map mantle structure with respect to a reference model such as PREM [47]: SAW24B16 [48], S362ANI [49], GyPSuM-S [50], S40RTS [51], Savani [52] and SEMUCB-WM1 [53]. As in ref. [54], the structure of the lower mantle is represented by cluster analysis of seismic wave velocities in tomographic models (only for the present-day) and mantle temperature in mantle flow models (present-day and back in time in 20 Myr increments), respectively. We use *k*-means clustering [55], a procedure that minimizes the variance in squared Euclidean distance between vectors to separate ~200,000 points equally-spaced on

Earth's surface into two clusters with similar variations between 1,000-2,800 km depths in either seismic velocity (for tomographic models, Fig. 3A,C) or mantle temperature (for mantle flow models, Fig. 3B, D).

Figure 3. Cluster analysis of tomographic and mantle flow models. A, B/ spatial distribution of two lower mantle regions revealed by k-means cluster analysis between 1,000 km and 2,800 km depths for **A/** tomographic model S40RTS [51] and **B/** mantle flow model Case 2. Coastlines are shown in black, and the grey lines indicate a value of five (solid) and a value of one (dashed) in a vote map of low-velocity regions from five S-wave tomographic models [56]. **C, D/** depth profiles of clustered properties in each cluster: **C/** velocity profiles in high-velocity (fast, in blue) and low-velocity (slow, in red) regions for S40RTS and **D/** temperature profiles in low-temperature (cold, in blue) and high-temperature (hot, in red) regions for Case 2. The solid curves are the mean, and the transparent envelopes are the associated standard deviation.

2.5. Quantifying model success

2.5.1.1. Success for the present-day: match with tomographic models

We assess the success of mantle flow models in reproducing the present-day structure of the mantle by quantifying the match between cluster maps for tomographic models and flow models as in ref. [54]. The accuracy $Acc = (TP+TN)/A$ involves the area of true positive (TP, orange in Fig. 4) match (where high-temperature clusters from a given flow model intersect slow-velocity clusters from a given tomographic model), and the area of true negative (TN, grey in Fig. 4) match (where low-temperature clusters from a given flow model intersect fast-

velocity clusters from a given tomographic model) over the total area *A*. The sensitivity (sometimes called recall or true positive rate) $S = TP/(TP+FN)$ involves the true positive area (*TP*, orange in Fig. 4) and the false negative (*FN,* blue in Fig. 4) area (where low-temperature clusters from a given flow model intersect slow-velocity clusters from a given tomographic model).

Figure 4. Spatial match of lower mantle structure between mantle flow and tomographic models with respect to tomographic model S40RTS. A/ Intersection of mantle structure between S40RTS and mantle flow model Cases 1-6 as indicated. Orange (true positive) indicates low-velocity tomography and high-temperature regions, grey (true negative) indicates high-velocity tomography and low-temperature regions, green (false positive) indicates high-velocity tomography and high-temperature regions and blue (false negative) indicates low-velocity tomography and low-temperature regions. **B/** As in **A** but for tomographic model S40RTS and other tomographic models as indicated. Orange (true positive) indicates low-velocity regions for both models, grey (true negative) indicates high-velocity regions for both models, green (false positive) indicates high-velocity regions for S40RTS and low-velocity regions for other models and blue (false negative) indicates low-velocity regions for S40RTS and high-velocity regions for other models.

2.5.2. Success and predictions in the past: distance between reconstructed kimberlite eruptions and model high-prospectivity areas

We reconstruct the two considered sets of craton geometries back in time and compute the intersections of these cratons with either present-day LLSVPs (assumed to be stationary and

rigid back in time) mapped from cluster-analysis of the six considered S-wave tomographic models, or time-dependent basal mantle structures mapped with cluster-analysis in the seven considered flow models. These intersections are considered regions where kimberlites are likely to occur (kimberlite high-prospectivity regions). We compute time-dependent prospectivity maps as the distance from these intersections in the mantle reference frame, as well as relative prospectivity maps that summarise prospectivity over a given period in the plate frame of reference; in the latter, prospectivity is obtained by summing intersections between cratons and basal mantle structures over time, with values of one within intersections (prospective area at a given time) and zero outside. "Relative" prospectivity is obtained by normalising the result with respect to the highest.

To evaluate the success of models in predicting past kimberlite eruptions, we measure the distance between each reconstructed kimberlite and the nearest intersection between a craton and basal mantle structure. These minimum distances, cumulated over time, are summarised in one empirical distribution function for each combination of cratons and basal mantle structures, and the median and average minimum distances from these EDFs are used as indicators of model success.

3. Results

3.1. Area of basal mantle structures in tomographic and mantle flow models

The fractional area f_a of basal mantle structures affects the match between mantle flow models and tomographic models, as well as the minimum distances between kimberlites and prospectivity areas because models with larger f_a result in larger prospectivity areas, leading to smaller minimum distances to kimberlites.

For LLSVPs mapped as slow-velocity clusters from tomographic models, f_a ranges between 0.31 and 0.39 to the exception of GyPSuM-S for which the area is 0.52 (Fig. 5). In the mantle flow models, the fractional area covered by the high-temperature cluster changes from $f_a = 1.0$ in the initial condition until it stabilizes within a range (Fig. 5A). Over time, sinking slabs reduce the fractional area of the high-temperature cluster. This effect is most pronounced in cases with slabs initially inserted down to 1,000 km depth (Cases 1, 2 and 6 in Fig. 5A). In all cases, the area of basal mantle structures stabilises by about 640 Ma (360 Myr into the model run), which is about twice the time it takes for slabs to sink to the deep mantle in these models [57]. For this reason, we analyse models for the last 640 Myr.

Figure 5. Fractional area covered by hot/slow basal mantle structures in mantle flow and tomographic models. A/ Evolution of the fractional area of basal mantle structures f_a in flow models (blue circles and lines) and for tomographic models (red diamonds at present-day). The dashed vertical line is at 640 Ma. **B/** Fractional area of basal mantle structures averaged since 640 Ma ($\overline{f_a}$, blue diamonds with standard deviation shown as error bars on the left-hand-side panel, sorted by decreasing $\overline{f_a}$), compared to present-day f_a for tomographic models (open green diamonds on the right-hand-side panel, sorted by increasing f_a).

In the flow models, the fractional area covered by basal mantle structures f_a also depends on the buoyancy of the basal layer: it is larger for a denser basal layer ($\overline{f_a} \approx 0.47$ for Case 3 with $\delta\rho_b = +2.05\%$) and smaller for a less dense basal layer ($\overline{f_a} \approx 0.26$ for cases Cases 1, 4 and 5 with $\delta\rho_b = +1.02\%$). Case 2 with $\delta\rho_b = +1.43\%$ results in $\overline{f_a} \approx 0.37$ which is consistent with tomographic models ($0.31 < f_a < 0.39$). For a given density of the basal layer, f_a is larger for M21 ($\overline{f_a} \approx 0.31$ for Case 6) than for M21NNR ($\overline{f_a} \approx 0.26$ for cases Cases 1, 4

and 5), due to the different location of subduction zones with different reference frames (Fig. 4A).

3.2. Match between predicted mantle structure and tomographic models

The shape and size of basal thermochemical structures differ between model cases. Flow models that better match present-day tomographic models can be extrapolated back in time with more confidence, because the structure of the lower mantle is shaped by the history of subduction. To evaluate how the mantle structure from flow models matches that from tomographic models, we compute the accuracy $Acc = (TP+TN)/A$ and sensitivity $S = TP/(TP+FN)$ between tomographic models and between each mantle flow model and tomographic model (Fig. 6). The accuracy is a global match, whereas the sensitivity is a match between imaged and predicted and slow/hot basal mantle structure.

Figure 6. Quantitative match between predicted and imaged basal mantle structures. Match between hot basal mantle structures predicted by flow models and LLSVPs in seismic tomography models as mapped between 1,000 km and 2,800 km depths using cluster analysis. Left-hand-side panels show the sensitivity and right-hand-side panels the accuracy. In the top panels, quantities are derived between six flow models and six tomographic models, and in the bottom panels they are derived between six tomographic models.

Overall, the match between tomographic models ($0.58 < S < 0.86$ and $0.76 < Acc < 0.88$, excluding auto-correlation for which both scores are equal to one, Fig. 6) is

better than the match between mantle flow and tomographic models ($0.33 < S < 0.74$ and $0.54 < Acc < 0.78$). This result is expected since tomographic models are generally similar to one another in the way they were built, whereas the forward mantle flow models that cover one billion years of tectonic history are completely independent from tomographic models. Within tomographic models, the largest average sensitivities and accuracies are obtained for tomographic models S40RTS and SEMUCB-WM1. In contrast, GyPSuM-S appears to be an outlier for which the accuracy and sensitivity are systematically lower than for other tomographic models, because f_a is greater in that tomographic model (Figs 4, 5 and 6). Amongst mantle flow models, the sensitivity is largest between Case 1, 4 and 5 and S40RTS, Savani and S362ANI; it is also relatively large for Case 2. The accuracy is largest for the same mantle flow models but with S40RTS and SEMUCB-WM1. The accuracy and sensitivity are lowest for Case 6 that is based on reconstruction M21, in which net lithospheric rotation present in the surface boundary conditions induces wholesale motion of the lower mantle and basal mantle structures [35]. For this reason, we focus on M21NNR in the following.

There is a trade-off between f_a and S and Acc: model cases in which the basal layer is less dense (Cases 1, 4 and 5) result in larger S and Acc (match to tomographic models), however in these cases f_a is smaller than in tomographic models. Case 2, for which f_a is comparable to tomographic models and Acc and S are reasonably large, is our preferred mantle flow model case. In the following, we first illustrate results for Case 2 and tomographic models S40RTS (which is the best tomographic model based on Acc and S) before summarising results for all models.

3.3. Reconstructing kimberlites, cratons and basal mantle structures back to 640 Ma

As an example, we reconstruct kimberlites occurrences from ref. [15], cratons from ref. [29], and use basal mantle structures in S40RTS and Case 2 back to 640 Ma (Fig. 7 and S1 Animation). S40RTS better captures reconstructed southern African kimberlites than Case 2 back to 80 Ma. However, before 80 Ma, Case 2 better captures the peak of South African kimberlite activity at 100 Ma. All flow models based on reconstruction M21NNR capture the southern African kimberlite blooms back to around 200 Ma, as illustrated by comparing S40RTS and Case 2 at 120 Ma, 160 Ma and 240 Ma (Fig. 7). S40RTS and Case 2 are both generally consistent with kimberlite eruptions on the Siberian craton back to 360 Ma. S40RTS does not match the kimberlite records in Antarctica, North China, and much of the kimberlite magmatism in Siberia; these locations are captured by Case 2 (Fig. 7).

Figure 7. Evolution of the location of slow/hot basal mantle structures and kimberlite eruptions. Kimberlite eruption locations (stars) sampled in 20 Myr increments from ref. [15] and craton shapes (white transparent polygons) from ref. [29], both reconstructed at 120 Ma (**A, E**), 160 Ma (**B, F**), 240 Ma (**C, G**) and 360 Ma (**D, H**) with reconstruction M21NNR. The background shows cluster maps with basal mantle structures in pink for tomographic model S40RTS (**A-D**) and for mantle flow model Case 2 (**E-H**). The intersections of white and pink areas are high-prospectivity regions for kimberlites. Labels: **A**/ “Si”: Siberia; **B**/ “An”: Antarctica; **C**/ “Afr”: Africa, “Au”: Australia, “Ca”: Canada ; **D**/ “N.Am”: North America, “N.Ch”: North China, “S.Afr”: South Africa.

Neither S40RTS nor Case 2 capture kimberlite magmatism in the Americas at 120 Ma. However, Case 2 better captures kimberlite occurrences than S40RTS in southern Africa and Antarctica at that time. ~~S40RTS encompasses the Western Australian kimberlite occurrence at that time whereas Case 2 does not (Fig. 7A, E).~~ In contrast, Case 2 captures kimberlite occurrences in the Northern Territory of Australia at 240 Ma and 360 Ma, whereas S40RTS does not (Fig. 7C, G).

At 160 Ma (Fig. 7B, F), both tomographic and flow models capture some north-eastern Canadian kimberlites although neither models matches the North American kimberlites until 170 Ma; as in previous work [7]. At 240 Ma, S40RTS captures Siberian and north-eastern Canadian kimberlites (Fig. 7C), and Case 2 captures kimberlites in Siberia, Africa, and Australia (Fig. 7G). Both models capture some Siberian kimberlites at 360 Ma (Fig. 7D, H).

3.4. Global kimberlite prospectivity maps

3.4.1. Global prospectivity maps in the mantle frame of reference

We next present the time-dependent distance to polygons defined by the intersection of reconstructed cratons and basal mantle structures as mapped using cluster analysis. In these kimberlite prospectivity maps, the distance is zero within the intersection and increases away from the intersections (Fig. 8 and S2 Animation).

Figure 8. Global kimberlite prospectivity maps and kimberlite eruptions through time. Distance to the intersection between basal mantle structures and reconstructed cratons from [29] at 120 Ma (**A, E**), 160 Ma (**B, F**), 240 Ma (**C, G**) and 360 Ma (**D, F**) for tomographic model S40RTS (**A-D**) and mantle flow model Case 2 (**E-H**). Cratons from ref. [29] are shown

as transparent white polygons, and kimberlite eruptions sampled in 20 Myr increments from ref. [15] are shown as stars; both are reconstructed using M21NNR. Labels: **A**/ "Gr": Greenland, "Si": Siberia; **B**/ "An": Antarctica, "N.Eu": Northern Europe; **C**/ "Afr": Africa, "Au": Australia, "Ca": Canada, "In": India ; **D**/ "N.Am": North America, "N.Ch": North China, "S.Afr": South Africa, "S.Am": South America, "S.Ch": South China.

Prospectivity maps for S40RTS and Case 2 both indicate the southern African cratons, eastern Canada, Greenland, northern Europe, the eastern African coast, and the west coasts of India and Madagascar as high-prospectivity areas for kimberlite magmatism over the last 240 Myr. Again, the predicted prospectivity before 80 Ma in southernmost Africa is smaller for S40RTS than for Case 2 (S2 Animation). S40RTS predicts lower prospectivity than Case 2 for South America, Australia and Antarctica at 360 Ma (Fig. 8D, H). Predicted prospectivity is low for terranes to the north of the Tethys Ocean and in particular for South China and North China between 120 Ma and 240 Ma in both models despite a kimberlite occurrence in North China at 240 Ma (Fig. 8C, G). S40RTS predicts increased prospectivity at 360 Ma for South China and North China, whereas Case 2 does not (Fig. 8D, H). Since reconstructed kimberlites are generally within or close to high prospectivity areas (Fig. 8), we next quantify the distance of reconstructed kimberlites to high prospectivity areas to evaluate the success of different models.

3.4.2. Global prospectivity maps in the plate frame of reference

We next present relative prospectivity over a given period in the plate frame of reference (Fig. 9A-D). This shows that for reconstruction M21NNR, the largest predicted prospectivity is in central and northern Africa for S40RTS (Fig. 9A, C), whereas it is in southern Africa for

Case 2 (Fig. 9B, D). This result is similar over 320 Myr (Fig. 9A, B) and 640 Myr (Fig. 9C, D). Because more regions are at the intersection between cratons and basal mantle structures when a longer model duration is considered, the area of prospective regions is larger over 640 Myr (Fig. 9C, D) than over 320 Myr (Fig. 9A, B). Overall, the relative prospectivity at kimberlite locations is greater for Case 2 than for S40RTS (Fig. 9E, F): the peak in the number of kimberlites is for a relative prospectivity of about 0.3 for S40RTS (median 0.27 and mean 0.24) and about 0.55 for Case 2 (median 0.50 and mean 0.41) over 320 Ma (Fig. 9E); over 640 Ma (Fig. 9F) the peak is at ~0.15 for S40RTS (median 0.20 and mean 0.22) and ~0.7 for Case 2 (median 0.41 and mean 0.40). We note that there are also significant peaks at low-prospectivity (<0.3), and that these peaks are higher for Case 2 than for S40RTS (Fig. 9E, F).

Figure 9. Predicted relative kimberlite prospectivity. A-D/ Global kimberlite relative prospectivity maps since 320 Ma (**A-B**) and 640 Ma (**C-D**) for tomographic model S40RTS (**A, C**) and Case 2 (**B, D**). Kimberlites from ref. [15] are shown as black circles for the last 320 Ma (**A-B**) and 640 Ma (**C-D**). **E-F/** Distribution of relative prospectivity at kimberlite locations shown in **A-D** for S40RTS and Case 2 since 320 Ma (**E**) and 640 Ma (**F**).

3.5. Distance between kimberlites and predicted kimberlite high-prospectivity areas

3.5.1. Empirical Distribution Functions

We sample the distance to the closest kimberlite high-prospectivity region (referred to as minimum distance) at each reconstructed kimberlite locations (Fig. 8) and report results cumulated over time as Empirical Distribution Functions (EDFs) for each combination of basal mantle structures and craton shapes and for the last 640 Ma (Fig. 10). These EDFs show the

likelihood that a given proportion of kimberlites falls within a given minimum distance from a kimberlite high-prospectivity region. Thus, EDFs with smaller minimum distances reflect cases for which reconstructed kimberlites are closer to predicted kimberlite high-prospectivity areas, which indicates more successful models that better account for the kimberlite record. Models with a greater likelihood that distances are equal to zero are also more successful. As expected, distances are smallest for tomographic model GyPSuM-S and flow model Case 3 (Fig. 10) because these models result in larger basal mantle structures (Figs 4, 5).

Figure 10. Distances between kimberlite eruption locations and high-prospectivity areas. Empirical Distribution Functions (EDFs) of cumulative minimum distances since 640 Ma between reconstructed kimberlites (sampled in 20 Myr increments) and predicted high prospectivity areas for cratons from ref. [29] reconstructed using M21NNR, for all six tomographic models, and mantle flow models Cases 1-5 (that are based on M21NNR).

3.5.2. Metrics from Empirical Distribution Functions

To summarise EDFs, we report the mean minimum distance and associated standard deviation for each distribution, as well as the median minimum distance (distance for which the likelihood is equal to 0.5) (Fig. 11). The mean and median minimum distances confirm a link between reconstructed kimberlite eruption locations, reconstructed craton shapes, and basal mantle structure locations: median minimum distances indicate that 50% of kimberlites are within ~500 km of high-prospectivity areas for all models (Fig. 11A, C) and mean minimum distances are less than 1500 km (Fig. 11B, D). Mean and median minimum distances are similar for mantle flow models and tomographic models (Fig. 11), suggesting that mobile and deforming basal mantle structures are as consistent with the kimberlite record as stationary and rigid ones. This first order result, as well as trends between models, are similar over 320 Ma

(Fig. 11A, B) and over 640 Ma (Fig. 11C, D). Distances are largest (least favourable) for cases Cases 4 and 5, in which slabs are initially inserted down to 550 km depth and the density of the basal layer is intermediate (+1.02%; Table 1). Standard deviations are large for tomographic model S362ANI and flow model cases Cases 1, 4 and 5 (Fig. 11), reflecting more spread distributions (Fig. 10) due to smaller f_a (Fig. 4B). In contrast, mean and median distances are smallest for tomographic model GyPSuM-S and mantle flow Case 3, however these two cases do not match other tomographic models (Fig. 6) due to large basal mantle structures (Fig. 5B). Interestingly, mantle flow model Case 2 results in relatively small minimum distances (Fig. 11) as well as being compatible with tomographic models (Figs 5 and 6). For Case 2, 50% of kimberlites are within ~100 km of high-prospectivity areas over 320 Ma (Fig. 11A), and 50% of kimberlites are within ~200 km of high-prospectivity areas over 640 Ma (Fig. 11C). When craton shapes from ref. [37] are used, trends are similar, and mean and median distances are approximately 1.5 times as large as with craton shapes from ref. [29] (Figs 11 and 12). When reconstruction M21 (in which the net rotation of the lithosphere is not removed) is used, mean and median distances are approximately twice as large than with reconstruction M21NNR for tomographic models (Figs 11 and 13).

Figure 11. Results for craton shapes from ref. [29] and reconstruction M21NNR. Metrics from EDFs for craton shapes from [29] and reconstruction M21NNR for mantle flow and tomographic models since 320 Ma (**A-B**) and 640 Ma (**C-D**). **A, C**/ Median and **B, D**/ average minimum distances with one standard deviation as error bar. In each panel, mantle flow model cases are to the left and tomographic models to the right of the dashed vertical line.

Figure 12. Results for craton shapes from and reconstruction M21NNR. Same as Fig. 11 but for craton shapes from ref. [37] and reconstruction M21NNR.

Figure 13. Results for craton shapes from ref. [29] and reconstruction M21. Same as Fig. 11 but for for craton shapes from ref. [29] and reconstruction M21 (in which the net rotation of the lithosphere is not removed).

4. Discussion

4.1. Model predictions: Regions of potential interest for large-scale kimberlite exploration

The global kimberlite prospectivity maps presented in this study (Figs 7 and 8) may be used for global-scale kimberlite exploration, placing them at the base of the hierarchy of scale-dependent targeting processes [58]. Cratons that have repeated intersections with model slow or hot clusters are more likely to have undergone kimberlite magmatism and are therefore of potential interest for large-scale kimberlite exploration. Prospectivity maps for S40RTS and Case 2 capture regions of prominent kimberlite magmatism such as Africa [59] and Siberia [60]. Case 2 also predicts some kimberlite activity in northwest Australia, Greenland, northeast Canada and Northern Europe (Figs 8 and 9D). Interestingly, Case 2 indicates Antarctica as a location of kimberlite prospectivity for the last 640 Ma (Fig. 9D), although there is only one known instance of kimberlite magmatism on the continent [61].

4.2. Mobility of basal mantle structures

Previous work has been shown that the reconstructed location of ~1,100 kimberlites over the last 320 Myr are within ~1,650 km of the edge of the African LLSVP as imaged by SMEAN at 2,800 km depth [7]. Interestingly, ~240 kimberlite occurrences from the Slave Province of Canada were more distant from the edge of the African LLSVP. Nevertheless, reconstructed locations of 25 LIPs erupted since 297 Ma were also close to the edge of

LLSVPs, leading to the hypotheses that 1/ mantle plumes arise from LLSVPs, and 2/ that LLSVPs could have been stationary over the last 300 Myr, and possibly longer [7, 62].

Here, we assumed that a relationship between basal mantle structures and kimberlites exists, and we considered two main scenarios: one in which lower mantle structures are mobile (represented by mantle flow models) and one in which lower mantle structures are stationary over time (represented by tomographic models). Overall, we found that minimum distances between kimberlites and high-prospectivity areas for kimberlites are similar for the two scenarios (Fig. 11-13), and that mobile basal structures better predict the abundance of kimberlites in southern Africa than stationary basal mantle structures (Figs 7-9). This suggests that kimberlites are as close to mobile basal structures as they are to stationary basal mantle structures, and that there is no need to hypothesise that basal mantle structures have been stationary over time, which is consistent with the mobility of tectonic plates, subduction zones [29, 33] and mantle plumes [63, 64] over time.

4.3. Limitations

Our global kimberlite prospectivity maps are based on the assumption that kimberlites are associated with deep mantle upwelling above basal mantle structures. However, kimberlite eruptions may also occur due to partial melting at the base of the lithosphere or above subducting slabs [65] and can be emplaced through weak zones within the lithosphere or close to plate boundaries [66]. Indeed, kimberlites from the Slave Province that are not closely associated with the African LLSVP (Figs. 7-9) could be associated to the history of subduction under North America [7]. Even though upwelling of hot mantle from the deep Earth may provide the source of heat leading to kimberlite eruption, the physical processes that could link basal mantle structures [>2000 km deep; 19] and kimberlite eruption most often from ~200 km depth [3] are not yet firmly established.

The presented reconstructions of past mantle flow do not predict kimberlite eruptions. Modelling kimberlite eruptions requires a horizontal resolution of a few hundred meters, modelling two-phase flow (melt and solid) and considering viscosity contrast over 26 orders of magnitude between the melt with viscosity 0.01 Pa s [67] and the lithosphere with viscosity about 1 x 10^{24} Pa s. The presented time-dependent global mantle flow models achieve a horizontal resolution of 50 km, viscosity contrast over three orders of magnitude, and do not consider melting. Achieving kilometre-scale resolution is possible in instantaneous global mantle flow models [68] but remains prohibitively expensive in time-dependent models. Viscosity contrasts of six orders of magnitude have been achieved in global mantle flow models [69], however these models ~~were~~ are not paleogeographically-driven so could not be as readily compared to the geological record as the models presented here. Modelling magmatic processes in 3D global spherical geometry remains a frontier.

Our results are sensitive to the density of the basal mantle structures, which is a key control on their fractional area (f_a). In our preferred Case 2, basal structures were intrinsically 1.43% denser than surrounding mantle. This density is comparable to previous models [e.g. 28, 70], and with that inferred from Earth's free oscillations [up to 1.7%; 71], however, it is larger than those estimated from tidal tomography 0.5% [22]. Further research is required to determine the density of basal mantle structures since seismically-filtered mantle flow models [72] and tomography including Stoneley modes [73] suggest that they could be purely thermal.

We used the only available full-plate tectonic reconstruction extending back to 1 Ga, and considered the original reconstruction [M21; 29] and a version of the reconstruction in which net rotation of the lithosphere was removed (M21NNR). The latter is appropriate for mantle flow modelling since net rotation of the lithosphere is thought to arise from lateral viscosity variations within the mantle [34, 74]. Indeed, imposed lithospheric net rotation results in induced net rotation of the lower mantle and results in a poor match of lower mantle

structures with tomographic models [35]. Nevertheless, lithospheric net rotation is unlikely to be equal to zero; it could be between 0.023°/Myr [75] and 0.26°/Myr [76]. Further work is required to develop new tectonic reference frames for paleogeographically-constrained global mantle flow models.

5. Conclusion

We assumed that a link exists between upwelling above basal mantle structures and kimberlite magmatism to investigate the relationships between kimberlite eruptions, cratons, and lower mantle structure over the last 640 Myr. To investigate the hypothesis that basal mantle structures might have been stationary over time, we considered lower mantle structure to be either stationary and given by tomographic models, or mobile and predicted by reconstructions of past mantle flow over the past billion years. We showed that the present-day structure of the lower mantle predicted by flow models is compatible with that imaged by tomographic models. We created prospectivity maps for kimberlites by computing the time-dependent intersection between reconstructed craton shapes and lower mantle structures. We measured the distance of reconstructed kimberlite eruption locations to high-prospectivity regions, which confirmed that kimberlites are generally close to high-prospectivity regions, although this result depends on the reference frame being used and craton shapes considered for reconstruction; for the preferred mantle flow model case 50% of kimberlites were within ~100 km of high-prospectivity areas over 320 Ma and within ~200 km of high-prospectivity areas over 640 Ma. We found that mobile basal mantle structures better predicted the peak in kimberlite eruption in southern Africa at ~100 Ma than stationary mantle structures. Overall, these results suggest that mobile basal mantle structures are as consistent with kimberlite eruptions as stationary ones. We therefore favour a model in which basal mantle structures

have been mobile over time, which is consistent with observations and models suggesting that tectonic plates, subduction zones and mantle plumes have also been mobile over time.

Acknowledgements

NF and OFB were supported by Australian Research Council grant LP170100863. This research was undertaken with the assistance of resources from the National Computational Infrastructure (NCI), which is supported by the Australian Government. This research was supported by the Australian Government's National Collaborative Research Infrastructure Strategy (NCRIS), with access to computational resources provided by the National Computational Infrastructure (NCI) through the National Computational Merit Allocation Scheme. Access to NCI was partly supported by resources and services from the University of Wollongong (UOW). Key model results and scripts are available at https://doi.org/10.5281/zenodo.5760115.

References

1. Gurney JJ, Helmstaedt HH, Richardson SH, Shirey SB. Diamonds through Time. Economic Geology. 2010;105(3):689-712. doi: 10.2113/gsecongeo.105.3.689.
2. Clifford TN. Tectono-metallogenic units and metallogenic provinces of Africa. Earth and Planetary Science Letters. 1966;1(6):421-34.
3. Anfilogov VN, Khachai YV. Origin of the kimberlite diamond-bearing lithosphere of cratons. Dokl Earth Sc. 2013;451(2):814-7. doi: 10.1134/S1028334X13080096.
4. Hoggard MJ, Czarnota K, Richards FD, Huston DL, Jaques AL, Ghelichkhan S. Global distribution of sediment-hosted metals controlled by craton edge stability. Nature Geosci. 2020;13(7):504-10.
5. Aryasova OV, Khazan YM. "Clifford's Rule" and the geodynamics of kimberlite magmatism. Geofizicheskiy Zhurnal. 2013;35(6):101-13. doi: 10.24028/gzh.0203-3100.v35i6.2013.116453.
6. Shchipansky AA. Subduction geodynamics in Archean and formation of diamond-bearing lithospheric keels and early continental crust of cratons. Geotecton. 2012;46(2):122-41. doi: 10.1134/S0016852112020057.
7. Torsvik TH, Burke K, Steinberger B, Webb SJ, Ashwal LD. Diamonds sampled by plumes from the core–mantle boundary. Nature. 2010;466(7304):352-5. doi: 10.1038/nature09216.
8. O'Reilly SY, Griffin WL, Djomani YHP, Morgan P. Are lithospheres forever? Tracking changes in subcontinental lithospheric mantle through time. GSA today. 2001;11(4):4-10.
9. Sparks RSJ, Brown RJ, Field M, Gilbertson M. Kimberlite ascent and eruption. Nature. 2007;450(7172):E21-E. doi: 10.1038/nature06435.
10. Wilson L, Head Iii JW. An integrated model of kimberlite ascent and eruption. Nature. 2007;447(7140):53-7. doi: 10.1038/nature05692.
11. Zahirovic S, Müller RD, Seton M, Flament N. Tectonic speed limits from plate kinematic reconstructions. Earth and Planetary Science Letters. 2015;418:40-52.
12. Harte B. Diamond formation in the deep mantle: the record of mineral inclusions and their distribution in relation to mantle dehydration zones. Mineral mag. 2010;74(2):189-215. doi: 10.1180/minmag.2010.074.2.189.
13. Smith EM, Shirey SB, Richardson SH, Nestola F, Bullock ES, Wang J, et al. Blue boron-bearing diamonds from Earth’s lower mantle. Nature. 2018;560(7716):84-7. doi: 10.1038/s41586-018-0334-5.
14. De Wit M, Bhebe Z, Davidson J, Haggerty SE, Hundt P, Jacob J, et al. Overview of diamond resources in Africa. 2016.
15. Tappe S, Smart K, Torsvik T, Massuyeau M, de Wit M. Geodynamics of kimberlites on a cooling Earth: Clues to plate tectonic evolution and deep volatile cycles. Earth and Planetary Science Letters. 2018;484:1-14. doi: 10.1016/j.epsl.2017.12.013.
16. Ault AK, Flowers RM, Bowring SA. Synchroneity of cratonic burial phases and gaps in the kimberlite record: Episodic magmatism or preservational bias? Earth and Planetary Science Letters. 2015;410:97-104. doi: 10.1016/j.epsl.2014.11.017.
17. Kampunzu A, Lubala RT. Magmatism in extensional structural settings: the Phanerozoic African plate: Springer Science & Business Media; 2012.
18. Becker TW, Boschi L. A comparison of tomographic and geodynamic mantle models. Geochem Geophys Geosyst. 2002;3(1).
19. Garnero EJ, McNamara AK. Structure and dynamics of Earth's lower mantle. Science. 2008;320(5876):626-8.

20. Torsvik TH, van der Voo R, Doubrovine PV, Burke K, Steinberger B, Ashwal LD, et al. Deep mantle structure as a reference frame for movements in and on the Earth. Proceedings of the National Academy of Sciences. 2014;111(24):8735-40. doi: 10.1073/pnas.1318135111.
21. Ni S, Tan E, Gurnis M, Helmberger D. Sharp sides to the African superplume. Science. 2002;296(5574):1850-2.
22. Lau HC, Mitrovica JX, Davis JL, Tromp J, Yang H-Y, Al-Attar D. Tidal tomography constrains Earth's deep-mantle buoyancy. Nature. 2017;551(7680):321-6.
23. Burke K, Steinberger B, Torsvik TH, Smethurst MA. Plume Generation Zones at the margins of Large Low Shear Velocity Provinces on the core–mantle boundary. Earth and Planetary Science Letters. 2008;265(1-2):49-60. doi: 10.1016/j.epsl.2007.09.042.
24. Austermann J, Kaye BT, Mitrovica JX, Huybers P. A statistical analysis of the correlation between large igneous provinces and lower mantle seismic structure. Geophysical Journal International. 2014;197(1):1-9.
25. Davies D, Goes S, Sambridge M. On the relationship between volcanic hotspot locations, the reconstructed eruption sites of large igneous provinces and deep mantle seismic structure. Earth and Planetary Science Letters. 2015;411:121-30.
26. Courtillot V, Davaille A, Besse J, Stock J. Three distinct types of hotspots in the Earth's mantle. Earth and Planetary Science Letters. 2003;205(3-4):295-308. doi: 10.1016/S0012-821X(02)01048-8.
27. Bunge H. Time Scales and Heterogeneous Structure in Geodynamic Earth Models. Science. 1998;280(5360):91-5. doi: 10.1126/science.280.5360.91.
28. McNamara AK, Zhong S. Thermochemical structures beneath Africa and the Pacific Ocean. Nature. 2005;437(7062):1136-9. doi: 10.1038/nature04066.
29. Merdith AS, Williams SE, Collins AS, Tetley MG, Mulder JA, Blades ML, et al. Extending full-plate tectonic models into deep time: Linking the neoproterozoic and the phanerozoic. Earth-Science Reviews. 2021:103477.
30. Merdith AS, Collins AS, Williams SE, Pisarevsky S, Foden JD, Archibald DB, et al. A full-plate global reconstruction of the Neoproterozoic. Gondwana Research. 2017;50:84-134. doi: 10.1016/j.gr.2017.04.001.
31. Domeier M. A plate tectonic scenario for the Iapetus and Rheic oceans. Gondwana Research. 2016;36:275-95.
32. Domeier M. Early Paleozoic tectonics of Asia: Towards a full-plate model. Geoscience Frontiers. 2018;9(3):789-862. doi: 10.1016/j.gsf.2017.11.012.
33. Young A, Flament N, Maloney K, Williams S, Matthews K, Zahirovic S, et al. Global kinematics of tectonic plates and subduction zones since the late Paleozoic Era. Geoscience Frontiers. 2019;10(3):989-1013. doi: 10.1016/j.gsf.2018.05.011.
34. Coltice N, Gérault M, Ulvrová M. A mantle convection perspective on global tectonics. Earth-science reviews. 2017;165:120-50.
35. Rudolph ML, Zhong S. History and dynamics of net rotation of the mantle and lithosphere. Geochem Geophys Geosyst. 2014;15(9):3645-57.
36. Griffin W, O'Reilly SY, Afonso JC, Begg G. The composition and evolution of lithospheric mantle: a re-evaluation and its tectonic implications. Journal of Petrology. 2009;50(7):1185-204.
37. Artemieva IM. Global 1°×1° thermal model TC1 for the continental lithosphere: Implications for lithosphere secular evolution. Tectonophysics. 2006;416(1-4):245-77. doi: 10.1016/j.tecto.2005.11.022.
38. Zhong S, Zhang N, Li Z-X, Roberts JH. Supercontinent cycles, true polar wander, and very long-wavelength mantle convection. Earth and Planetary Science Letters. 2007;261(3-4):551-64. doi: 10.1016/j.epsl.2007.07.049.

39. Bower DJ, Gurnis M, Flament N. Assimilating lithosphere and slab history in 4-D Earth models. Physics of the Earth and Planetary Interiors. 2015;238(0):8-22. doi: http://dx.doi.org/10.1016/j.pepi.2014.10.013.
40. Williams S, Wright NM, Cannon J, Flament N, Müller RD. Reconstructing seafloor age distributions in lost ocean basins. Geoscience Frontiers. 2021;12(2):769-80.
41. Gurnis M, Turner M, Zahirovic S, DiCaprio L, Spasojevic S, Müller RD, et al. Plate tectonic reconstructions with continuously closing plates. Computers & Geosciences. 2012;38(1):35-42. doi: http://dx.doi.org/10.1016/j.cageo.2011.04.014.
42. Chopelas A, Boehler R. Thermal expansivity in the lower mantle. Geophysical Research Letters. 1992;19(19):1983-6.
43. Steinberger B, Calderwood AR. Models of large-scale viscous flow in the Earth's mantle with constraints from mineral physics and surface observations. Geophysical Journal International. 2006;167(3):1461-81.
44. Ricard Y, Richards M, Lithgow-Bertelloni C, Le Stunff Y. A Geodynamic Model of Mantle Density Heterogeneity. J Geophys Res. 1993;98(B12):21895-21,909.
45. Hernlund JW, Houser C. On the statistical distribution of seismic velocities in Earth's deep mantle. Earth and Planetary Science Letters. 2008;265(3-4):423-37.
46. Bunge H-P, Richards MA, Lithgow-Bertelloni C, Baumgardner JR, Grand SP, Romanowicz BA. Time scales and heterogeneous structure in geodynamic Earth models. Science. 1998;280(5360):91-5.
47. Dziewonski AM, Anderson DL. Preliminary reference Earth model. Physics of the earth and planetary interiors. 1981;25(4):297-356.
48. Mégnin C, Romanowicz B. The three-dimensional shear velocity structure of the mantle from the inversion of body, surface and higher-mode waveforms. Geophysical Journal International. 2000;143(3):709-28. doi: 10.1046/j.1365-246X.2000.00298.x.
49. Kustowski B, Ekström G, Dziewoński AM. Anisotropic shear-wave velocity structure of the Earth's mantle: A global model. J Geophys Res. 2008;113(B6):B06306. doi: 10.1029/2007JB005169.
50. Simmons NA, Forte AM, Boschi L, Grand SP. GyPSuM: A joint tomographic model of mantle density and seismic wave speeds. J Geophys Res. 2010;115(B12):B12310. doi: 10.1029/2010JB007631.
51. Ritsema J, Deuss A, van Heijst HJ, Woodhouse JH. S40RTS: a degree-40 shear-velocity model for the mantle from new Rayleigh wave dispersion, teleseismic traveltime and normal-mode splitting function measurements: S40RTS. Geophysical Journal International. 2011;184(3):1223-36. doi: 10.1111/j.1365-246X.2010.04884.x.
52. Auer L, Boschi L, Becker TW, Nissen-Meyer T, Giardini D. Savani : A variable resolution whole-mantle model of anisotropic shear velocity variations based on multiple data sets. J Geophys Res Solid Earth. 2014;119(4):3006-34. doi: 10.1002/2013JB010773.
53. French SW, Romanowicz BA. Whole-mantle radially anisotropic shear velocity structure from spectral-element waveform tomography. Geophysical Journal International. 2014;199(3):1303-27. doi: 10.1093/gji/ggu334.
54. Flament N, Williams S, Müller RD, Gurnis M, Bower DJ. Origin and evolution of the deep thermochemical structure beneath Eurasia. Nat Commun. 2017;8(1):14164. doi: 10.1038/ncomms14164.
55. MacQueen J, editor Some methods for classification and analysis of multivariate observations. Proceedings of the fifth Berkeley symposium on mathematical statistics and probability; 1967: Oakland, CA, USA.
56. Lekic V, Cottaar S, Dziewonski A, Romanowicz BA. Cluster analysis of global lower mantle tomography: A new class of structure and implications for chemical heterogeneity. Earth and Planetary Science Letters. 2012;357:68-77.

57. Flament N. Present-day dynamic topography and lower-mantle structure from palaeogeographically constrained mantle flow models. Geophysical Journal International. 2019;216(3):2158-82. doi: 10.1093/gji/ggy526.
58. Hronsky JM, Groves DI. Science of targeting: definition, strategies, targeting and performance measurement. Australian Journal of Earth Sciences. 2008;55(1):3-12.
59. Field M, Stiefenhofer J, Robey J, Kurszlaukis S. Kimberlite-hosted diamond deposits of southern Africa: A review. Ore Geology Reviews. 2008;34(1-2):33-75. doi: 10.1016/j.oregeorev.2007.11.002.
60. Ashchepkov IV, Logvinova AM, Ntaflos T, Vladykin NV, Kostrovitsky SI, Spetsius Z, et al. Alakit and Daldyn kimberlite fields, Siberia, Russia: Two types of mantle sub-terranes beneath central Yakutia? Geoscience Frontiers. 2017;8(4):671-92. doi: 10.1016/j.gsf.2016.08.004.
61. Yaxley GM, Kamenetsky VS, Nichols GT, Maas R, Belousova E, Rosenthal A, et al. The discovery of kimberlites in Antarctica extends the vast Gondwanan Cretaceous province. Nat Commun. 2013;4(1):2921. doi: 10.1038/ncomms3921.
62. Torsvik TH, Steinberger B, Ashwal LD, Doubrovine PV, Trønnes RG. Earth evolution and dynamics—a tribute to Kevin Burke. Canadian Journal of Earth Sciences. 2016;53(11):1073-87.
63. Hassan R, Müller RD, Gurnis M, Williams SE, Flament N. A rapid burst in hotspot motion through the interaction of tectonics and deep mantle flow. Nature. 2016;533(7602):239-42.
64. Tarduno J, Bunge H-P, Sleep N, Hansen U. The bent Hawaiian-Emperor hotspot track: Inheriting the mantle wind. Science. 2009;324(5923):50-3.
65. Giuliani A, Pearson DG. Kimberlites: from deep earth to diamond mines. Elements: An International Magazine of Mineralogy, Geochemistry, and Petrology. 2019;15(6):377-80.
66. Jelsma H, Barnett W, Richards S, Lister G. Tectonic setting of kimberlites. Lithos. 2009;112:155-65.
67. Kono Y, Kenney-Benson C, Hummer D, Ohfuji H, Park C, Shen G, et al. Ultralow viscosity of carbonate melts at high pressures. Nat Commun. 2014;5(1):1-8.
68. Stadler G, Gurnis M, Burstedde C, Wilcox LC, Alisic L, Ghattas O. The dynamics of plate tectonics and mantle flow: from local to global scales. Science. 2010;329(5995):1033-8.
69. Coltice N, Husson L, Faccenna C, Arnould M. What drives tectonic plates? Science advances. 2019;5(10):eaax4295.
70. Trim S, Lowman J, Butler S. Improving mass conservation with the tracer ratio method: application to thermochemical mantle flows. Geochem Geophys Geosyst. 2020;21(2):e2019GC008799.
71. Ishii M, Tromp J. Normal-mode and free-air gravity constraints on lateral variations in velocity and density of Earth's mantle. Science. 1999;285(5431):1231-6.
72. Davies D, Goes S, Lau H. Thermally dominated deep mantle LLSVPs: a review. The Earth's heterogeneous mantle. 2015:441-77.
73. Koelemeijer P, Deuss A, Ritsema J. Density structure of Earth's lowermost mantle from Stoneley mode splitting observations. Nat Commun. 2017;8(1):1-10.
74. Ricard Y, Doglioni C, Sabadini R. Differential rotation between lithosphere and mantle: a consequence of lateral mantle viscosity variations. J Geophys Res Solid Earth. 1991;96(B5):8407-15.
75. Becker TW. On the effect of temperature and strain-rate dependent viscosity on global mantle flow, net rotation, and plate-driving forces. Geophysical Journal International. 2006;167(2):943-57.

76. Conrad CP, Behn MD. Constraints on lithosphere net rotation and asthenospheric viscosity from global mantle flow models and seismic anisotropy. Geochem Geophys Geosyst. 2010;11(5).

Supporting Information

S1 Animation. Evolution of the location of slow/hot basal mantle structures and kimberlite eruptions. Kimberlite eruption locations (stars) sampled in 20 Myr increments from ref. [15] and craton shapes (white transparent polygons) from ref. [29], reconstructed in 20 Myr increments since 640 Ma. The background shows cluster maps with basal mantle structures in pink for tomographic model S40RTS (left-hand-side) and for mantle flow model Case 2 (right-hand-side). The intersections of white and pink areas are high-prospectivity regions for kimberlites.

S2 Animation. Global kimberlite prospectivity maps and kimberlite eruptions through time. Global kimberlite prospectivity maps in 20 Myr increments since 640 Ma for tomographic model S40RTS (left-hand-side) and mantle flow model Case 2 (right-hand-side) showing the distance to the intersection between basal mantle structures and cratons from ref. [29] reconstructed using M21NNR. Cratons are shown as transparent white polygons and kimberlite eruption locations sampled in 20 Myr increments from ref. [15] are shown as stars.
